# Flow Rate and Water Depth Alters Biomass Production and Phytoremediation Capacity of *Lemna minor*

**DOI:** 10.3390/plants11162170

**Published:** 2022-08-21

**Authors:** Neil E. Coughlan, Éamonn Walsh, Roger Ahern, Gavin Burnell, Rachel O’Mahoney, Holger Kuehnhold, Marcel A. K. Jansen

**Affiliations:** 1 School of Biological, Earth and Environmental Sciences, University College Cork, Distillery Fields, North Mall, T23 TK30 Cork, Ireland; 2 Environmental Research Institute, University College Cork, T23 XE10 Cork, Ireland; 3 Department of Ecology, Leibniz Centre for Tropical Marine Research (ZMT), 28359 Bremen, Germany

**Keywords:** biomass production, duckweed, Lemnaceae, nutrient recovery, phytoremediation

## Abstract

Given its high biomass production, phytoremediation capacity and suitability as a feedstock for animal and human nutrition, duckweeds are valuable multipurpose plants that can underpin circular economy applications. In recent years, the use of duckweeds to mitigate environmental pollution and valorise wastewaters through the removal of excess nitrogen and phosphate from wastewaters has gained considerable scientific attention. However, quantitative data on optimisation of duckweed performance in phytoremediation systems remain scant. In particular, a mechanistical understanding of how physical flows affect duckweed growth and remediation capacity within vertical indoor multi-tiered bioreactors is unknown. Here, effects of flow rate (0.5, 1.5 or 3.0 L min^−1^) and medium depth (25 mm or 50 mm) on *Lemna minor* biomass production and phytoremediation capacity were investigated. Results show that flow rates and water depths significantly affect both parameters. *L*. *minor* grew best at 1.5 L min^−1^ maintained at 50 mm, corresponding to a flow velocity of 0.0012 m s^−1^. The data are interpreted to mean that flow velocities should be low enough not to physically disturb duckweed but still allow for adequate nutrient mixing. The data presented will considerably advance the optimisation of large-scale indoor (multi-tiered, stacked), as well as outdoor (pond, lagoon, canal), duckweed-based remediation of high nutrient wastewaters.

## 1. Introduction

The per capita availability of nutritional food and clean water is expected to substantially decrease in coming decades [1,2]. An anticipated global population growth of up to 9.8 billion people by 2050, coupled with the ongoing progression of climate change and increased levels of environmental pollution, will jeopardise food and water security worldwide [2,3]. Furthermore, given the array of environmental problems caused by fossil fuel consumption, there is an urgent need to identify and develop alternative energy sources such as sustainable biofuels, e.g. [4]. Accordingly, there is an urgent need to improve food, water and fuel security through the development of innovative novel crops and cropping systems that are sustainable and limit the consumption of finite resources, such as nutrients and water, and the generation of waste [3,5,6]. Closed-loop production systems can be used to minimise resource inputs and waste outputs in accordance with the principles of a circular economy approach, whereby the long-term retention and reuse of resources is prioritised over the addition of new raw materials [5,7]. For example, phytoremediation can be used to remove excess nutrients from wastewaters, facilitating re-use of water, and recycling of nutrients in a variety of industrial applications, e.g. [8,9].

As small floating aquatic plants, duckweed species (Lemnaceae) generally display rapid growth on nutrient-rich waters, as well as relatively high tolerances to a range of pollutants [10,11,12]. Further, the rapid growth of duckweed species is matched by a rapid uptake of plant nutrients from the medium e.g. [13,14]. Hence, duckweed species can be effectively used to remediate a variety of nutrient-rich wastewaters, including diluted livestock farm manure [9,14,15], aquaculture wastewater [16] or dairy processing wastewater [17,18]. Notably, duckweed biomass can be used to produce biofuel (e.g., ethanol and methane) [19]. Additionally, given their nutritionally desirable composition of both amino acids and poly-unsaturated fatty acids [10,20,21], duckweeds are also increasingly studied for their potential as a nutritious biomass for livestock and human consumption [10,22]. As such, duckweed-based water remediation can contribute to a recycling of plant nutrients in accordance with the principles of the circular economy [5]. Accordingly, innovative duckweed cropping systems could be used for environmental remediation and wastewater valorisation [23].

To date, duckweed biomass production, and phytoremediation of wastewaters, has generally occurred in ponds, lagoons, or canal-based systems [23,24]. These systems tend to be either outdoors or encapsulated within structures such as glasshouses or polytunnels [23]. In many regions, these systems can be relatively cheap to build and maintain and are often capable of accommodating 100s–1000s of litres of wastewater input per day. However, the large-scale cultivation of duckweed in pond-based systems can have a large spatial requirement [23]. As an alternative, indoor growth systems composed of multi-tiered, vertically stacked layers can be used to increase duckweed biomass production and phytoremediation capacity per unit area of land [23,25]. This may be especially pertinent in urban and semi-urban areas, where infrastructure is limited by horizontal rather than vertical space availability. Multi-tiered, indoor systems operated in controlled environments have additional potential benefits, such as optimised cultivation, improved year-round growth, and operation under sterile conditions, thus avoiding contamination by bacteria, viruses, fungi, algae, and invertebrates, e.g. [23,24,26].

There are considerable knowledge gaps concerning the basic operating parameters of both outdoor and indoor duckweed-based remediation systems. In particular, the effects of flow rate on duckweed biomass production and phytoremediation are largely unknown. To date, most studies have tended to assess performance of static or low flow systems with only periodic or extremely slow entry and exit of wastewater media (e.g., 1.0–1.5 L d^−1^) [23] or have focused on wetland communities rather than strictly duckweed cultivation, but see [27]. Flow rates are critical determinants of the performance of duckweed-based systems, as they determine nutrient supply, mixing and the residence time of the medium. Sufficiently high flow rates relative to both the volume of the duckweed-based system and the duckweed surface cover, are required to avoid nutrient depletion. However, an adequate residence/retention time is required to facilitate optimal remediation. Despite this, an approach whereby flow rates and residence times are exclusively based on physico-chemical considerations is incomplete. In the case of duckweed bioreactors, plant biological aspects also need to be considered. Duckweeds are adapted to still or slow-flowing waters [28], however, the maximum flow rates tolerated by duckweeds are unknown. High flow rates may result in the formation of thick layers of piled-up duckweed and/or inhibit growth through physical disturbance. In this scenario, duckweed growth will be impeded, fronds will readily senesce and release nutrients back into the water column. Thus, it can be hypothesised that an optimal flow rate will exist that facilitates nutrient supply without impeding plant growth. Further, the water depth will co-determine the flow rate required to assure nutrient depletion. However, it is not clear whether the water depth will also directly impact on plant growth. Although duckweed has been observed to grow on just a few millimetres of water in the natural environment [29], in warm climates a sufficient depth of the water column may be required to prevent heat-stress in outdoor systems, e.g., >200 mm [30] and 500 mm [27].

In the present study, the effects of different flow rates (0.5, 1.5 or 3.0 L min^−1^) and medium depths (25 mm or 50 mm) on duckweed biomass production, plant health, and nitrogen and phosphorous uptake were quantified. Plants were cultivated on a standardised nutrient-rich medium within a simple, indoor, vertically stacked recirculating system. We hypothesised that greater flow rates would reduce biomass production, decrease plant health, and curtail nutrient uptake by plants due to increased physical disturbance by medium (i.e., greater velocity). The data will improve operation of duckweed-based remediation, duckweed biomass production, and food-production systems that seek to use duckweed technologies to implement closed-loop production systems.

## 2. Methods

### 2.1. Stock Cultivation

The duckweed strain used in this study was *Lemna minor* L. (Blarney strain, number 5500 in the Rutgers Duckweed Stock Cooperative database: www.ruduckweed.org (accessed on 1 May 2022) ). A non-axenic stock of *L. minor* was cultivated for the experiments. Stock plants were maintained on a nutrient-rich solution that consisted of tap water and commercially available nutrient additives: pH Perfect Grow (2 mL L^−1^) and pH Perfect Micro (2 mL L^−1^; Advanced Nutrients: Table S1). The stock culture was maintained indoors at an average light intensity of 150 μmol m^−2^ s^−1^ PAR (photosynthetically active radiation), at ~21 °C with a 16:8 hours light:dark photoperiod.

### 2.2. Experimental Design

**Figure 1:**
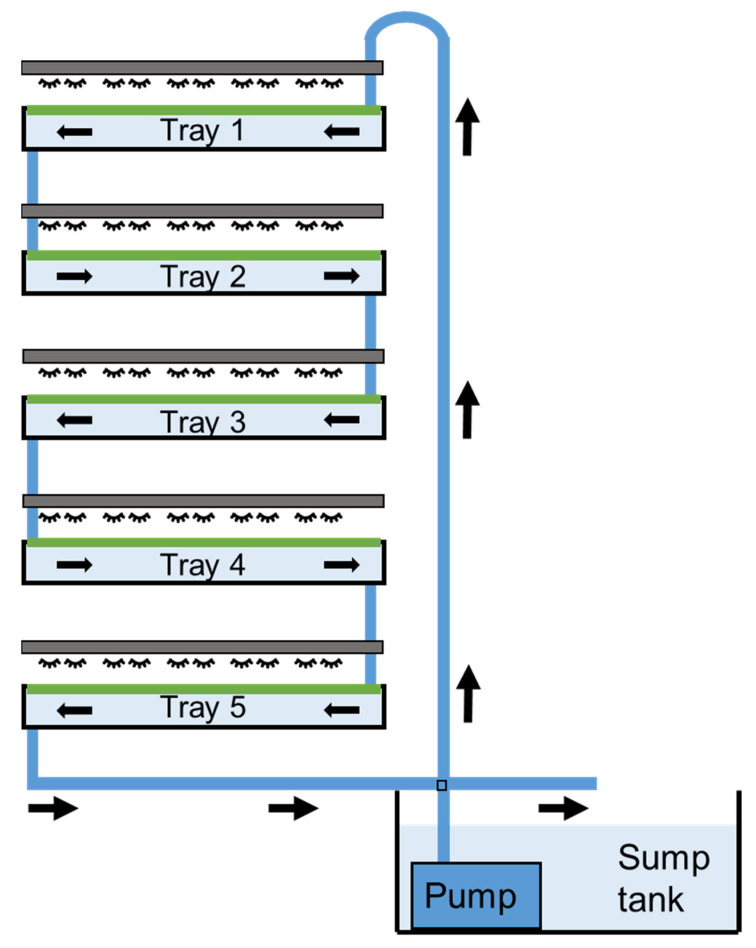
Schematic of the indoor, vertically stacked, 125-litre duckweed cultivation system.

The study of the effects of flow rate and water depth on *L. minor* was performed using an indoor, recirculating system. The system consisted of five vertically stacked trays (720 mm × 410 mm × 110 mm: length × width × height) and a sump tank (600 mm × 410 mm × 410 mm), operated at a combined capacity of 125 litres (Figure 1). Combined, the five trays had a surface area for duckweed growth of 1.48 m^2^. The trays were suspended within a supporting stainless-steel framework. A nutrient-rich solution was continuously pumped from the sump tank at floor level to the top tray (Tray 1: Rio Pump 1700, TAAM Inc.), all other trays were gravity fed. Medium exited the final tray (i.e., tray 5) to be deposited back into the sump tank. All piping had an internal diameter of 20 mm. The nutrient-rich solution consisted of commercially available nutrient additives and was prepared using distilled water (FloraGrow, 0.25 mL L^−1^; FloraMicro, 0.25 mL L^−1^: General Hydroponics: Table S1). Plants were maintained within the stacked system at an average light intensity of 150 μmol m^−2^ s^−1^ PAR using LED strip lighting (Neonica Growy, Neonica Polska Sp. z o.o.) at ~ 21 °C with a 16:8 h light:dark photoperiod. The 10 mm-wide LED strips used were uniformly installed every 20 mm to ensure an even distribution of light. Upon completion of a duckweed growing cycle the system was drained and all surfaces were thoroughly cleaned with hot water (≥60 °C).

Growth of duckweed within the vertically stacked system was assessed in relation to three flow rates (0.5, 1.5 or 3.0 L min^−1^) and two medium depths (25 mm or 50 mm). The highest flow rate (3.0 L min^−^^1^) was selected based on a preliminary assessment of the cultivation system. Flow rates greater than 3.0 L min^−1^ tended to cause the formation of overlapping duckweed layers that inhibit growth. Lower flow rates were based on the partial incremental reduction of the maximum flow rate. Flow rates were modulated by diverting excess medium back to the sump prior to entering tray 1, this was achieved with the use of a control-valve that could be manually adjusted to alter medium flow rates. The lowest medium depth (25 mm) was selected based on a preliminary assessment of the cultivation system, and simply doubled for assessment of the greater depth (50 mm). Depths of less than 25 mm tended to cause the formation of overlapping duckweed layers that inhibit growth, as the plants did not tend to spread evenly across the medium surface, even for the lowest flow rate. Medium depths were modulated by raising or lowering tray exit pipes, which were inserted perpendicular to the base of the trays. Calculated surface flow velocities are shown in Table 1. All possible treatment combinations were assessed in a factorial design (treatment replication: *n* = 3; see Table 1). To commence the experiment, a 60% surface cover of *L. minor* was established for each tray. To achieve this, 75 g (fresh weight) of *L. minor* was added to each tray. Fresh weight was achieved by draining the duckweed of excess water through a fine mesh sieve, and then blotting the duckweed dry using highly absorbent paper towels. Preliminary work using the imaging software Easy Leaf Area established that 75 g consistently provided for 60% surface cover of the trays. Easy Leaf Area software distinguishes duckweed frond surface cover from non-duckweed covered surface area. Each treatment lasted seven days. However, to avoid overcrowding, an intermediate harvest was employed on the third day to return the surface cover to 60% [as per 8]. At the intermediate harvest, all duckweed was gently removed using a flat sieve and weighed, with 75 g being returned to each tray.

### 2.3. Data Collection

Total biomass yield (fresh weight) was determined through summation of biomass detected on the third and seventh days relative to the initial biomass (75 g) for each tray. Further, the relative growth rate of biomass yield was determined using the formula below [31]:

(1) $$RGR=\frac{ln\frac{W_{2}}{W_{1}}}{\Delta T}$$

where *ln* is the natural log, *W*_1_ is the initial biomass, *W*_2_ is final biomass and Δ*T* is the length of the experiment. *RGR* was calculated from inoculation to intermediate harvest (day 0–3) and from the intermediate harvest to the final harvest (day 3–7). In addition, chlorophyll *a* fluorescence was measured using pulse amplitude modulated chlorophyll *a* fluorometry (WALZ Imaging fluorometer, Effeltrich, Germany). Chlorophyll *a* fluorescence was measured on days 0 and 7. Plants were adapted to dark conditions for 15 min immediately in advance of chlorophyll *a* fluorescence analysis. For assessment on day 0, three colonies (i.e., three plantlets of 3–4 fronds) were randomly selected from the stock of plants used to inoculate the stacked system. On day 7, three colonies were randomly taken from each tray. The measured values of these three colonies were averaged and treated as one replicate. The chlorophyll fluorescence analysis procedure used to measure fluorescence (F_0_) and maximum fluorescence (Fm) of the dark-adapted plant, as well as the maximum fluorescence (Fm’) and steady-state fluorescence (Ft) under light-adapted conditions, was in accordance with Walsh et al. [8,18]. The actinic light intensity (i.e., photosynthetically active light) used was of 186 μmol m^−2^ s^−1^. Subsequently, the maximum quantum efficiency of photosystem II (i.e., Fv/Fm), and the quantum efficiency of photosystem II under steady-state light conditions (i.e., Y(II)) were calculated according to the equations detailed by Maxwell and Johnson [32].

Plant biomass samples extracted from tray 1, 3 and 5 on day 7 were analysed for total nitrogen (TN) and total phosphate (TP) content (mg g^−1^), as was a representative sample of the plants used to inoculate all trays on day 0. Plants were dried and milled, and then digested at 420 °C for one hour with concentrated sulphuric acid and Kjeltab Cu/3.5 in a TECATOR 2040 Digestor. Digested samples were diluted to a volume of 250 mL using deionised water. The total nitrogen was determined using QuickChem IC + FIA flow injection analyzer (8000 series: Lachat Instruments). For plant TP content, the samples were acid digested and analysed using the ammonium molybdate method [33]. Absorbance was measured using a UV-Visible Recording Spectrophotometer (UV-160A: SHIMADZU Corporation).

Samples of the liquid medium were taken for TN and TP analysis on days 0 and 7. Day 0 samples were taken from the sump tank, while day 7 samples were separately taken from trays 1, 3, and 5. Unfiltered samples were used for both TN and TP analysis. TN samples were digested with potassium persulfate and boric acid in alkaline conditions, while TP samples were digested with ammonium persulfate in acidic conditions. Both sets of samples were digested in an autoclave at 120 °C for 30 min. The resulting total oxidised nitrogen was analysed by automated cadmium reduction method using Lachat Quikchem 8000 by Zellweger Analytics, Inc. Milwaukee, WI, USA [34], while the resulting phosphate was analysed manually using the Murphy and Riley Method [33].

The measurements of the concentrations of TN and TP were used to assess the nett TN and TP depletion, i.e., the decrease in concentration, over the 7-day period, for the entire 125-litre system. Nett nutrient removal was calculated based on nutrient depletion and the volume of the system. Removal was also normalised for the amount of duckweed present, yielding estimations for milligrams of N and *p* removed per m^2^ of *L. minor* per day (mg TN m^−2^ day^−1^; mg TP m^−2^ day^−1^) and milligrams of N and *p* removed per g of *L. minor* per day (mg TN g*^−^*^1^ day^−1^; mg TP g*^−^*^1^ day^−1^). For normalisation purposes, TN and TP removal was expressed relative to the starting amount of duckweed (i.e., 60% surface cover or 75 g per tray). The uptake of TN and TP by *L. minor* plants was also determined. To achieve this, the content of TN and TP per gram of dry duckweed biomass was converted to fresh weight using a conversion factor of 3.64% (% dry versus fresh weight). The conversion factor was determined on duckweed used to inoculate the 125-litre system by drying 50 g samples to a constant weight (60 °C; *n* = 5). The total amount of TN and TP taken up by plants was calculated by multiplying the plant TN and TP content by the total biomass present at the end of the 7-day cultivation period, and by subtracting the TN or TP content of the duckweed used to inoculate the system. An uptake rate was calculated by dividing by the duration of the experiment.

### 2.4. Data Analysis

Statistical analyses were conducted using R software (version R 4.1.2). All data were assessed for normality of residual distributions (Shapiro–Wilk test: library *psych*) and homoscedasticity of variances (Levene’s test: library *car*). Where data were found to be normally distributed (*p* > 0.05) with homoscedastic residuals (*p* > 0.05), general linear models (ANOVA) were used to analyse differences in biomass yield, RGR and nutrient depletion of the media. Logistic regression in the form of generalised linear models (GLM: *car*) were employed for non-normal data and/or heteroscedastic residuals (*p* < 0.05). The effects of flow rate, medium depth, and tray position (1–5) were considered in all models. A stepwise depletion approach was used to remove non-significant terms, while overall model significance was determined using likelihood ratio tests in all cases (*lmtest*). Where *p*-values were significant (α < 0.05), Tukey’s LSD adjustments for multiple pairwise comparisons were used for post-hoc analysis (*emmeans*) [35].

## 3. Results

### 3.1. Duckweed Growth

**Figure 2:**
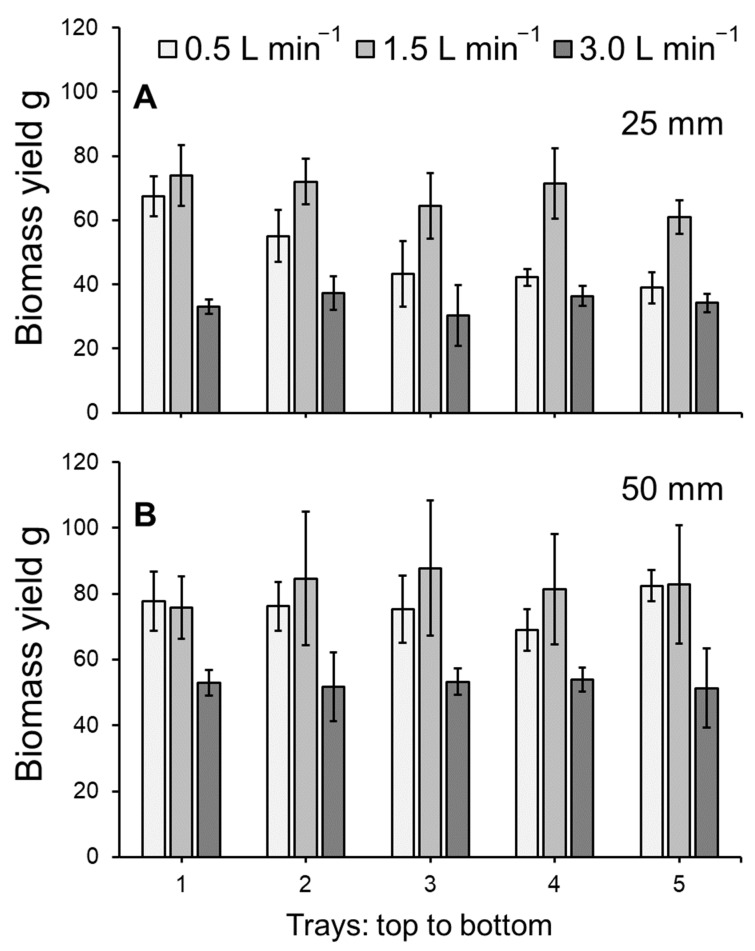
Mean (±SE) biomass yield (g) at medium depths of (**A**) 25 mm and (**B**) 50 mm for *Lemna minor* grown within an indoor, vertically stacked system. Plants were grown on hydroponics medium at three flow rates (0.5, 1.5 or 3.0 L min^-1^) and two medium depths (25 or 50 mm) over 7 days. See Figure 3 for a simplified model depiction with post-hoc analysis.

**Figure 3:**
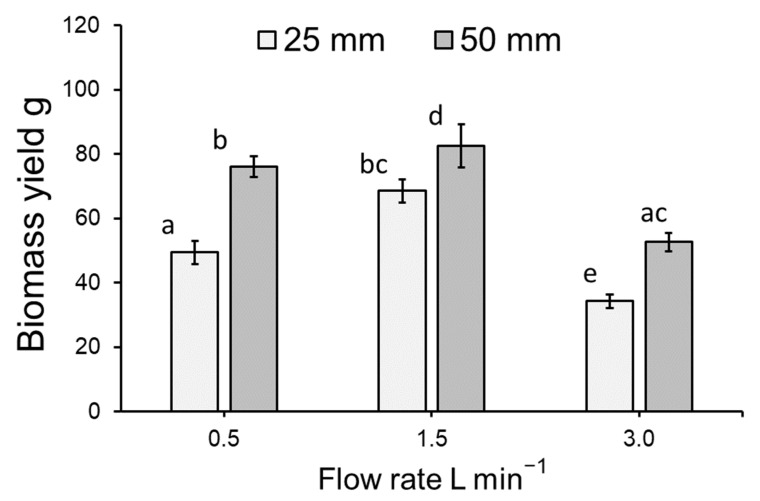
Mean (±SE) biomass yield (g) for *Lemna minor* grown within an indoor, vertically stacked system. As no effect of tray position was detected, trays have been pooled in this simplified model depiction. Letters show statistical similarity (*p* > 0.05). See Figure 2 for full model depiction.

The biomass yield of *L. minor* cultivated within the vertically stacked system was found to significantly differ across treatments of flow rates and medium depths (ANOVA: χ^2^ = 70.881, df = 3; *p* < 0.001: Figure 2A,B). As tray position did not have a significant effect on biomass yield (*p* > 0.05), data from different trays were pooled for a simplified model depiction (Figure 3). While both flow rate and medium depth had a significant effect on biomass yield (both *p* < 0.001), no interaction terms were detected amongst the model variables. Biomass yield was greatest for duckweed grown in a flow of 1.5 L min^−1^ with a medium depth of 50 mm compared to all other flow rate and depth combinations (all *p* < 0.05: see Figure 3).

**Figure 4:**
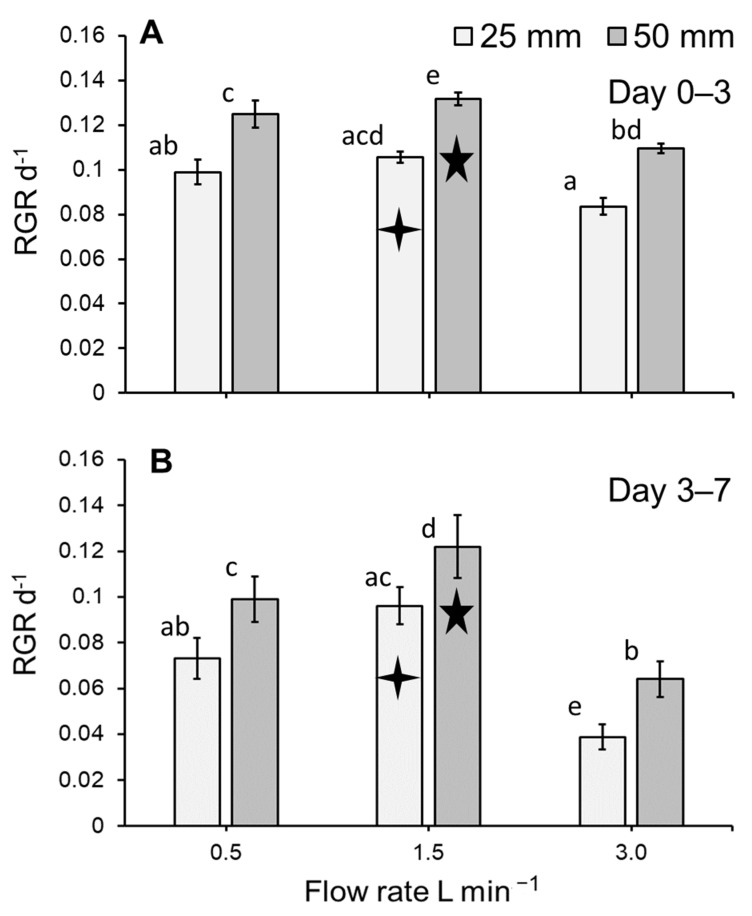
Mean (±SE) RGR (d^−1^) for *Lemna minor* grown within an indoor, vertically stacked system. RGR between inoculation and the intermediate harvest (day 0–3; (**A**) and the final harvest (day 3–7; (**B**) are shown. As no effect of tray position was detected, trays have been pooled in this simplified model depiction. Letters show statistical similarity within each harvest period separately (i.e., day 0–3 or 3–7), while symbols indicate similarity amongst the harvest periods within treatments (*p* > 0.05). Similarity amongst harvest periods is considered for within each treatment only (see Table 1).

Biomass yields were used to calculate RGR values to facilitate comparison with published literature. The RGR significantly differed for *L. minor* subjected to different treatments within the stacked system (ANOVA: χ^2^ = 108.98, df = 6; *p* < 0.0001: Figure 4A,B). Flow rate, medium depth, and assessment period (i.e., day 0–3 and day 3–7) had a significant effect on biomass yield (all *p* < 0.0001). RGR was generally greater for plants grown on a medium depth of 50 mm rather than 25 mm (*p* < 0.0001; Figure 4A,B). While RGR tended to be slightly lower for plants grown on a flow rate of 3.0 L min^−1^, this was not always statistically apparent (Figure 4A,B). A significant interaction effect on RGR was detected for flow and assessment period (*p* < 0.001). Furthermore, although not always statistically apparent, the RGR tended to be greater for plants grown in the period of days 0–3 compared to day 3–7 (Figure 4A,B).

### 3.2. Chlorophyll a Fluorometry

To determine the photosynthetic efficiencies of plants kept at different combinations of flow rate and water depth, chlorophyll *a* fluorescence was measured. In all cases, the maximum quantum efficiency (i.e., Fv/Fm) of photosystem II (PSII) for *L. minor* did not differ significantly for plants subjected to different treatments (GLM: χ^2^ = 3.4032, df = 17; *p* > 0.05). Mean Fv/Fm ranged from 0.63–0.77 across all treatments. Similarly, the quantum efficiency of PSII under steady-state light conditions (i.e., YII) for plants did not significantly differ (GLM: χ^2^ = 20.003, df = 17; *p* > 0.05). Mean YII ranged from 0.23–0.41 across all treatments.

**Figure 5:**
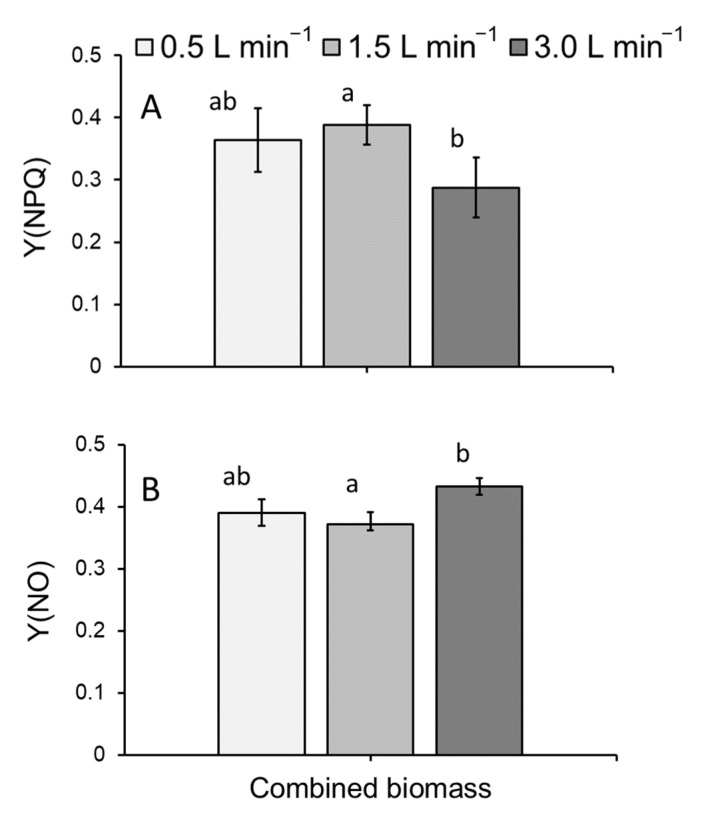
Mean (±SE) (**A**) Y(NPQ) and (**B**) Y(NO), for *Lemna minor* grown within an indoor, vertically stacked system. As no effect of depth or tray was detected, medium depths and tray positions have been pooled in this simplified model depiction. Letters show statistical similarity (*p* > 0.05).

Y(NPQ), the yield of regulated energy dissipation, was found to significantly vary (GLM: χ^2^ = 8.6834, df = 2; *p* < 0.05: Figure 5A). When pooled across depths and trays, a significantly lower Y(NPQ) was detected for *L. minor* cultivated at 3.0 L min^−1^ than for plants grown in a flow of 1.5 L min^−1^ (*p* < 0.05: Figure 5A). Finally, Y(NO), the yield of nonregulated energy dissipation, was also found to significantly vary (GLM: χ^2^ = 8.4029, df = 2; *p* < 0.05: Figure 5B). The combined Y(NO) of *L*. *minor* inoculated and cultivated at 3.0 L min^−1^ (pooled across depths and trays) was significantly greater than that of plants grown in a flow of 1.5 L min^−1^ (*p* < 0.01: Figure 5B).

### 3.3. Total Nitrogen Concentration of the Medium

**Figure 6:**
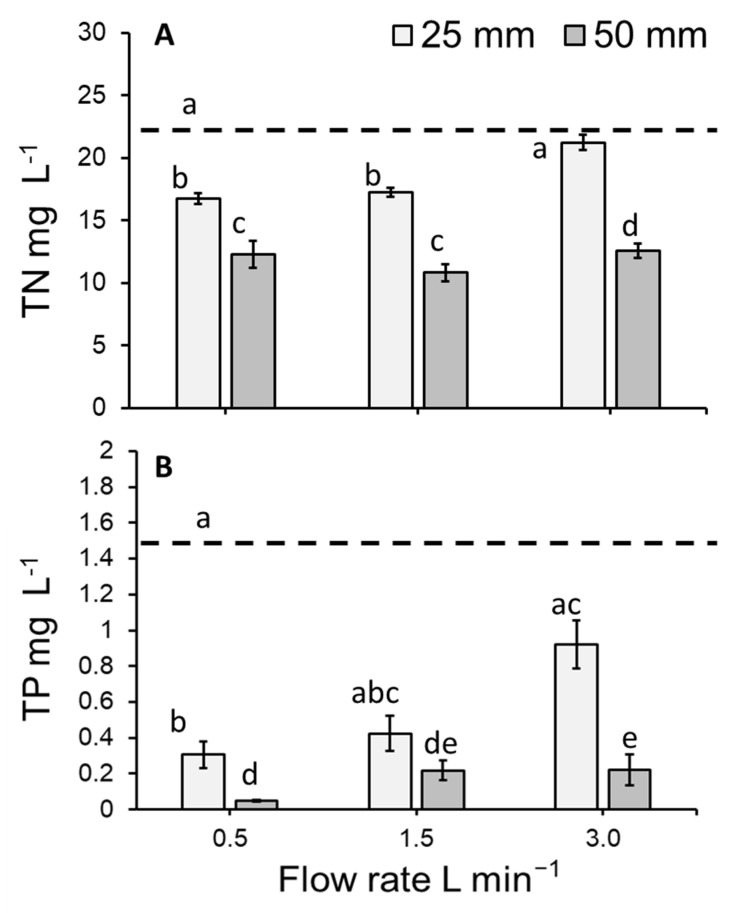
Mean (±SE) (**A**) total nitrogen concentration (TN mg L^−1^) and (**B**) total phosphorous concentration (TP mg L^−1^) of the hydroponics medium following the cultivate *Lemna minor* within an indoor, vertically stacked system. As no effect of tray was detected, trays have been pooled in this simplified model depiction. Letters show statistical similarity within each panel (*p* > 0.05). Dashed lines represent the initial concentration of TN (**A**) and TP (**B**).

The concentration of nitrogen (TN mg L^−1^) remaining in the medium within the vertically stacked system significantly differed amongst treatments (GLM: χ^2^ = 202.91, df = 7; *p* < 0.0001: Figure 6A). Flow rate (*p* < 0.05), depth and time point (i.e., initial concentration day 0 vs. final concentration day 7) were found to have a significant effect on TN concentration in the medium (both *p* < 0.0001). Compared to the initial concentration of TN on day 0 (i.e., 22.42 ± 0.35 mg L^−1^; mean ± SE), the concentration of nitrogen present in the medium was significantly reduced on day 7 for flow rates of 0.5 and 1.5 L min^−1^ (but not 3 L min^−1^) maintained at a depth of 25 mm (both *p* < 0.0001; Figure 6A). Using a water depth of 50 mm, the concentration of TN was significantly less on day 7 than on day 0 for all flow rates (all *p* < 0.0001; Figure 6A). At individual flow rates, a greater reduction in the total nitrogen concentration occurred at the 50 mm depth than at the 25 mm depth (all *p* < 0.001). At the 50 mm depth, the flow rates that showed the lowest concentration of nitrogen in the medium on day 7 (i.e., the greatest removal) were 0.5 and 1.5 L min^−1^ (i.e., *p* > 0.05: Figure 6A). A significant interaction effect was detected for flow and day (*p* < 0.001), as well as depth and day (*p* < 0.0001).

### 3.4. Total Phosphorous Concentration of the Medium

The concentration of phosphorous (TP mg L^−1^) in the medium within the vertically stacked system significantly differed amongst treatments (GLM: χ^2^ = 135.88, df = 7; *p* < 0.0001: Figure 6B). Time point was found to have a significant effect (*p* < 0.0001). Compared to the initial concentration of TP mg L^−1^ on day 0 (i.e., 1.46 ± 0.01 mg L^−1^), the concentration of phosphorous present in the medium was significantly reduced on day 7 for the flow and depth combination of 0.5 L min^−1^ and 25 mm (*p* < 0.0001; Figure 6B). Similarly, the TP concentration on day 7 was significantly lower than the initial concentration of TP for all flow rates maintained at a depth of 50 mm (all *p* < 0.0001). Within individual flow rates, a greater reduction of phosphorous occurred at the 50 mm depth than at the 25 mm depth (all *p* < 0.001). At the 50 mm depth, flow rates of 0.5 and 1.5 L min^−1^ showed the lowest concentration of phosphorous on day 7 (*p* > 0.05: Figure 6B). Although an effect of flow or depth was not statistically apparent (i.e., *p* > 0.05), a significant interaction effect was detected for flow and day (*p* < 0.001), as well as depth and day (*p* < 0.0001).

### 3.5. Total Nitrogen and Total Phosphorous Removal Per m^2^ of Lemna Minor

**Figure 7:**
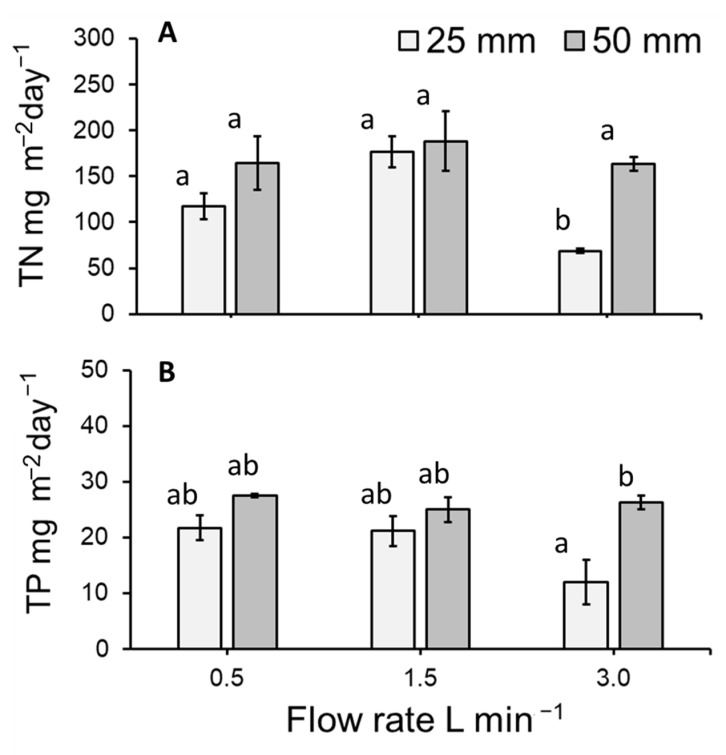
Mean (±SE) (**A**) TN removal per m^2^ of *Lemna minor* per day (mg m^−2^ day^−1^), (**B**) TP removal per m^2^ of *L. minor* per day (mg m^−2^ day^−1^) for plants cultivated on hydroponics medium at three flow rates and two medium depths over 7 days. Letters show statistical similarity within each panel (*p* > 0.05).

The total TN and TP removal from the system was calculated based on the depletion, i.e., decrease in the amount of TN and TP in the 125 litres of medium. Nutrient removal was normalised against the initial 60% surface cover or the initial inoculation mass of *L*. *minor* and recalculated as a rate by considering the duration of the treatment. The removal of mg TN m^−2^ day^−1^ from the medium significantly differed amongst treatments (GLM: χ^2^ = 26.211, df = 5; *p* < 0.0001: Figure 7A). Flow rate and depth (both *p* < 0.01) altered the rate of TN removal. A significant interaction effect was detected for flow and depth (*p* < 0.001). TN removal tended to not statistically differ among flow rates maintained at the same depth, nor between depths at a given flow rate. However, in comparison to all other treatment combinations, the removal of TN was significantly lower at a flow of 3 L min^−1^ when maintained at a depth of 25 mm (Figure 7A).

The removal of *p* (expressed as mg TP m^−2^ day^−1^) from the medium significantly differed amongst treatments (GLM: χ^2^ = 13.859, df = 5; *p* < 0.05: Figure 7B). Whilst depth altered the rate of TP removal (*p* < 0.05), no other significant effects were detected. TP depletion was similar among flow rates maintained at the same depth. Further, TP depletion did not statistically differ between depths kept at the same flow rate with the exception of 3 L min^−1^. The depletion of TP was significantly lower under the combined treatment of 3 L min^−1^ and 25 mm (Figure 7B).

### 3.6. Total Nitrogen and Total Phosphorous Uptake by Lemna Minor

**Figure 8:**
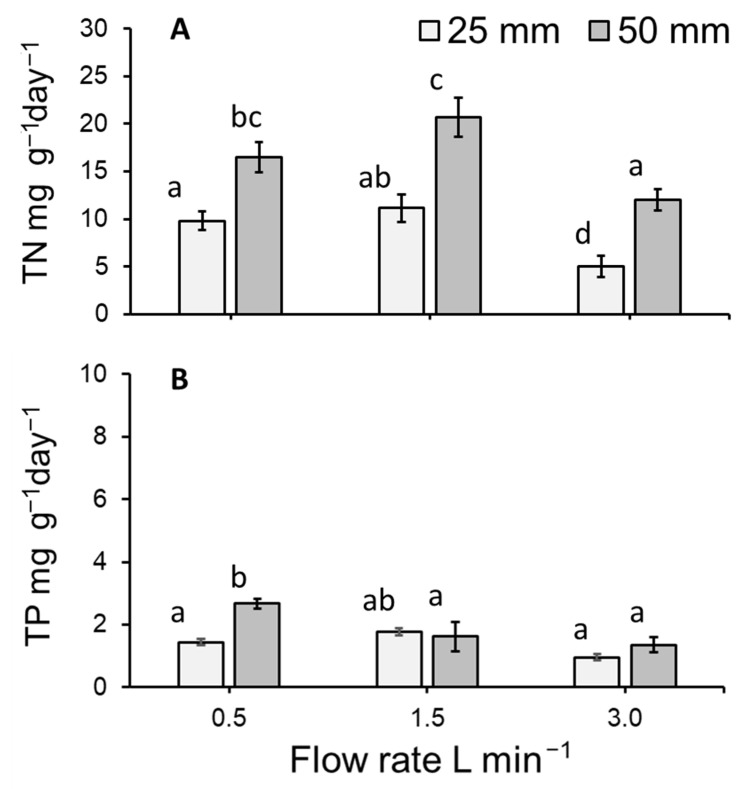
Mean (±SE) (**A**) TN and (**B**) TP uptake per gram of *Lemna minor* per day (mg g^−1^ day^−1^) for the biomass of plants cultivated on hydroponics medium at three flow rates and two medium depths over 7 days. As no effect of tray was detected, trays have been pooled in this simplified model depiction. Letters show statistical similarity within each panel (*p* > 0.05).

As a function of dry-weight biomass, moderate TN values of 2.5–3.9% and low TP values of 0.2–0.6% were detected in the harvested duckweed biomass. The nitrogen and phosphorous content (mg g^−1^) of dried *L. minor* biomass tended to be stable within treatments (Table S2). Calculated TN uptake rates (mg TN g^−1^ day^−1^) for the dried *L*. *minor* plants cultivated in the stacked system significantly differed amongst treatments (ANOVA: χ^2^ = 48.165, df = 3; *p* < 0.0001: Figure 8A). Flow rate and depth significantly altered the amount of TN taken up by plants (both *p* < 0.0001). No other significant effects were detected. The greatest TN uptake rate per gram of *L. minor* occurred at a depth of 50 mm for flows of 0.5 and 1.5 L min^−1^ (Figure 8A).

The uptake of TP (mg TP g^−1^ day^−1^) by plants cultivated within the stacked system significantly differed amongst treatments (ANOVA: χ^2^ = 27.023, df = 5; *p* < 0.0001: Figure 8B). Flow rate (*p* < 0.01) and depth (*p* < 0.05) significantly affected the rate of TP uptake. An interaction effect between flow rate and depth was also detected (*p* < 0.05). Flows 0.5 L min^−1^ and 1.5 L min^−1^ maintained at depths of 25 mm and 50 mm respectively, both displayed the greatest uptake of TP per gram of *L. minor* cultivated within the system (Figure 8B).

### 3.7. Fate of Total Nitrogen and Total Phosphorous within the System

**Figure 9:**
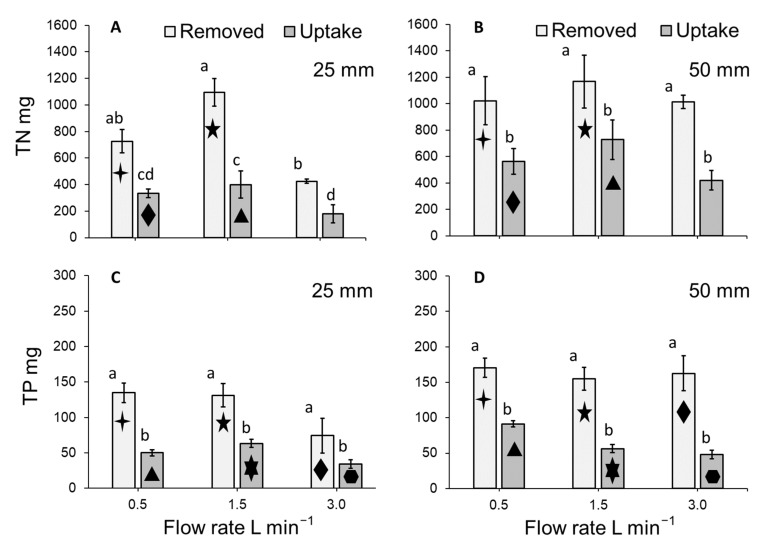
Mean (±SE) (**A**,**B**) total removal and total plant uptake of TN and (**C**,**D**) TP within the 125-litre stacked system. As no effect of tray was detected, trays have been pooled in this simplified model depiction. Letters show statistical similarity within each medium depth (i.e., within a panel), while symbols indicate similarity in relation to each flow rate across both medium depths (*p >* 0.05).

As the system was non-axenic, data on removal of nitrogen and phosphorous from the medium were compared with those concerning nutrient uptake by plants. The fate of TN differed amongst treatments (GLM: χ^2^ = 49.634, df = 7; *p* < 0.0001: Figure 9A,B). Flow rate and depth significantly altered the amounts of TN removed and taken up (*p* < 0.01 and *p* < 0.001, respectively). Interaction effects between TN fate (i.e., amount removed or taken up) and depth, as well as flow rate and depth were detected (both *p* < 0.05). In all cases, removal of TN from the medium was significantly greater than the amount taken up by plants cultivated at a depth of 25 mm (all *p* < 0.0001: Figure 9A), as well as 50 mm (all *p* < 0.01: Figure 9B). The amount of TN removed from the entire 125-litre system was the same amongst the flow rates within each depth category as was the amount of TN taken up by *L. minor*, with the exception of removal and depletion at 1.5 L min^−1^ and 3 L min^−1^ maintained at 25 mm (*p* > 0.05: Figure 9A,B). However, the amount of TN removed from the medium but not taken up by *L. minor* (i.e., the amount removed less the amount taken up) did not differ amongst treatments (GLM: χ^2^ = 10.554, df = 5; *p* = 0.06).

The fate of TP within the entire 125-litre system differed amongst treatments (GLM: χ^2^ = 29.18, df = 2; *p* < 0.0001: Figure 9C,D). Depth significantly altered the amount of TP removed and taken up (*p* < 0.01). No interactive effects were detected (*p* > 0.05). In all cases, removal of TP was significantly greater than the amount taken up by plants cultivated at either of the assessed depths (all *p* < 0.0001: Figure 9C,D). The amount of TP removed from the entire 125-litre system was the same for all flow rates maintained at either depth, as well as at a given flow rate between the assessed depths (Figure 9C,D). Similarly, TP uptake by *L. minor* cultivated within the stacked system was the same across all flow rates maintained at either depth, as well as between depths at a given flow rate (Figure 9C,D). The amount of TP removed from the medium but not taken up by *L. minor* (i.e., the amount removed less the amount taken up) did not differ amongst treatments (ANOVA: χ^2^ = 6.9684, df = 5; *p* > 0.05).

## 4. Discussion

The selection of flow rates and medium depths for duckweed biomass production and phytoremediation is a key challenge for the design and operation of both indoor and outdoor cultivation systems. Here, the growth, photosynthetic health, and nitrogen and phosphorous removal from the medium, and uptake by *L. minor* were quantified in relation to different combinations of flow rates and medium depths.

### 4.1. Duckweed Growth and Photosynthetic Health

The biomass yield and RGR of *L. minor* was reduced for plants maintained at a flow of 3 L min^−1^, especially at the shallower depth of 25 mm. Overall, mean RGR values (0.04–0.13 d^−1^) are lower than those reported in the literature for duckweed grown on an optimised medium (0.15–0.52 d^−1^) [36]. Nonetheless, RGR values recorded by the present study are in the range reported for duckweed grown on wastewater (0.03–0.25 d^−1^) [8,9,17]. Growth was fastest at the intermediate flow rate. The reduction in growth at the higher flow rate is conceivably caused by physical disturbance of the duckweed, especially in shallow water, with duckweed being forced away from the medium inlet and artificially overcrowded by the flow. Such physical disturbance is likely to be associated with water velocity, rather than flow rate. In this study, water velocity varied between 0.0004 and 0.0048 m s^−1^, with the reduction in depth from 50 mm to 25 mm resulting in a doubling of water velocity for any flow rate (see Table 1). Thus, negative effects of flow rate in duckweed bioreactors can be avoided by increasing water depth. Overall, the data on slower growth at a higher flow rate are in agreement with the common perception that duckweeds prefer still and slow-flowing waters. Yet, few studies present actual quantified data on water velocities that facilitate duckweed growth. A report by Derksen and Zwart [37] refers to velocities less than 0.1 m s^−1^, while a field study by Giblin et al. [33] indicated that a velocity of 0.095 m s^−1^ cannot be exceeded for a mixture of free-floating aquatic macrophytes, including various duckweed species: *L. minor*, *Spirodela polyrhiza*, and *Wolffia columbiana.* The high velocity observed by Giblin et al. [38], compared to the numbers reported in this study, may be explained by the focus on a maximal threshold for growth, as opposed to an optimal growth rate as aimed for in the current study. Indeed, in a field study by Janauer et al. [39], *L. minor* presence was associated with velocities of less than 0.05 m s^−1^. It remains to be seen through what mechanism higher flow rates impede duckweed growth. One possible scenario is the formation of overlapping layers of duckweed. Overcrowding has been associated with decreases in growth [40]. However, in the current study no overlapping layers of duckweed were observed.

At a lower flow rate (0.5 L min^−1^) we also observed small decreases in biomass accumulation and RGR, compared to growth at 1.5 L min^−1^. This reduced yield could be indicative of poor nutrient mixing within the system, especially when operated at a relatively shallow depth. The duckweed literature contains extensive data on maximum residence times required to either achieve (or avoid) nutrient depletion. Hydraulic residence times depend on nutrient concentrations, removal and uptake kinetics, and duckweed surface-to-tank volume ratios and therefore will vary across applications. However, commonly used residence times range from just a few days to more than a week [41,42,43]. As a result, it can be surmised that in the case of nutrient depletion, the last tray in the multi-tiered system would show very slow growth. However, this was not the case, as no significant tray effect on growth was noted across the flow rate and water depth combinations. Accordingly, nutrient depletion is considered unlikely as a cause of growth inhibition at the lowest flow rate. Accordingly, future work should consider fluid dynamics in greater detail, to ensure that the diffusion of nutrients within the geometric design of the system is adequate. Furthermore, for improved accuracy and precision of duckweed growth assessments, future work should consider the use of dry-weight biomass to minimize the variability associated with wet-weight measurements.

Photosynthetic activity measured as Fv/Fm, the maximum quantum yield of photosystem II, and Y(II), the effective quantum yield of photosystem II, were not affected by flow rate or water depth. However, an intriguingly small decrease in Y(NPQ) of plants grown at 3.0 L min^−1^, compared to 1.5 L min^−1^ was noted. This indicates an impairment in regulatory, non-photochemical quenching. In parallel, a small increase in Y(NO) is also noted. In combination with the decrease in Y(NPQ), this indicates a degree of plants stress [44]. One possible explanation for these data is that duckweed growth responded to the thigmotropic stimulus of water flow, which has been recorded for a range of aquatic plant species. Such a reorganisation of plant development may cause a decrease in growth [45].

### 4.2. Medium Concentration and Duckweed Removal (mg m^−2^ day^−1^) of Total Nitrogen and Total Phosphorous

Compared to the initial concentration, nitrogen (TN mg L^−1^) in the medium was similarly reduced at flow rates of 0.5 and 1.5 L min^−1^. However, the decrease in TN concentration was substantially less when a flow rate of 3.0 L min^−1^ was used. This effect was particularly strong in case of the shallow water depth of 25 mm. When nutrient depletion was normalised versus duckweed surface cover, TN removal per m^2^ of *L*. *minor* was broadly similar across all treatment combinations (mean: 117.22–188.39 mg TN m^−2^ day^−1^), except for the combination of the highest flow rate (3.0 L min^−1^) and shallow depth (25 mm) where nitrogen removal was substantially decreased. The observed TN removal rates were on the lower end of the wide range of values that can be found in the literature: 124–4400 mg TN m^−2^ d^−1^ [14,46,47,48,49]. Low TN removal rates may be due to the low TP concentration of the medium, which appears to slow both growth and TN removal. The lower TN removal rates at higher flow rates can be explained by relatively low duckweed growth rates observed under these conditions. Several authors have previously established links between growth and nutrient uptake, e.g. [8,13,18]. Thus, it appears that the negative effects of water velocity on plant growth are influencing plant phytoremediation potential.

The concentration of phosphorous (TP mg L^−1^) in the medium was also substantially reduced at most flow and depth combinations (up to 96.7%). The reduction in TP concentration was lowest at a flow rate of 3.0 L min^−1^ and a water depth of 25 mm. Normalised against plant surface area, TP removal rates varied between 11.99 and 27.51 mg TP m^−2^ day^−1^, with lowest removal at a flow of 3.0 L min^−1^ and a water depth of 25 mm. The observed TP removal rates were on the lower end of the wide range of values that can be found in the literature: 14–590 mg TP m^−2^ d^−1^ [14,46,47,48,49]. Low TP removal rates may be due to the low TP content of the medium. Furthermore, as noted for TN, low TP removal rates at higher flow rates can be explained by relatively low duckweed growth rates observed under these conditions. Although luxury uptake of phosphorous has been reported [50], in this study we noted a broad agreement between growth and phosphorous removal.

### 4.3. Duckweed Uptake (mg g^−1^ day^−1^) of Total Nitrogen and Total Phosphorous

Duckweed grown on the 50 mm water depth tended to uptake more TN than plants grown under shallower conditions, and these data generally match data on plant yield and RGR. However, the slight increase in growth at 1.5 L min^−1^ at 25 mm is, apparently, not matched by a similar increase of plant TN uptake. In general, TN content of duckweed can range from 0.8–7.8% when expressed as a function of dry-weight biomass [51]. In the present study, moderate TN values of 2.5–3.9% were detected. For TP, literature indicates that content generally ranges from 0.03–2.8% [51]. In the present study, TP values tended to be low (0.2–0.6%) and this may in part be a consequence of the employed medium being low in TP. Although *L. minor* can absorb large amounts of excess phosphorous into its tissues [52], almost no variations in phosphorous uptake were observed across flow rates.

### 4.4. Fate of Total Nitrogen and Total Phosphorous within the System

The quantities of TN and TP removed from the medium were substantially greater than the amounts taken up by *L. minor*. Between 36.5–62.3% and 29.5–53.5% of TN and TP removed, respectively, was taken up by duckweed biomass. The discrepancy between nutrient removal from medium and uptake in the duckweed plants has previously been detailed, e.g. [15]. The removal of TN and TP that is not directly accounted for by the nutrient content of duckweed biomass likely reflects the establishment of a biofilm consisting of microorganisms on the submerged surfaces of the system over the duration of the seven-day growing cycle [15] (*per*. *obs*. NEC and RA). In the present study, the amount of TN and TP removed from the medium but not taken up by *L. minor* (i.e., the amount removed less the amount taken up) did not vary amongst treatments, which indicates that the formation of a biofilm was independent of flow rate and water depth. The creation of this biofilm was likely due to the non-axenic experimental conditions and is a typical component part of most duckweed cultivation and/or phytoremediation systems.

## 5. Conclusions

In general, little information concerning the combined effects of flow rates and medium depths on duckweed biomass production and phytoremediation is available within the literature, and this paucity of quantitative data has impeded the optimisation of duckweed cultivation systems. The present study shows that flow rates and water depths can alter biomass production and phytoremediation capacity of *L. minor*. Plants grew best at an intermediate flow rate, which is congruent with the commonly accepted view that duckweeds prefer still and slow-flowing waters. It appears that optimal growth will need to be supported by a sufficient flow rate and medium depth to enable adequate nutrient mixing, but without causing physical disruption of Lemnaceae cultures. Accordingly, future work should consider the growth and phytoremediation of duckweed species by integrating a detailed understanding of fluid dynamics, uptake kinetics, plant disturbance tolerance and biofilm formation.

## Figures and Tables

**Table 1:** Treatment combinations of flow rates (0.5, 1.5 or 3.0 L min^−1^) and medium depths (25 mm or 50 mm). Calculated system velocities are shown.

Treatment	Flow Rates (L min^−1^)	Medium Depth (mm)	Velocity (m s^−1^)
1	0.5	25	0.0008
2	1.5	25	0.0024
3	3.0	25	0.0048
4	0.5	50	0.0004
5	1.5	50	0.0012
6	3.0	50	0.0024

## Data Availability

The data presented in this study are available on request from the corresponding author.

## Supplementary Materials

The following supporting information can be downloaded at: https://www.mdpi.com/article/10.3390/plants11162170/s1, Table S1: Water chemistry analysis for a single sample of the commercially available nutrient additives prepared using distilled water; Table S2: Mean proportional change of nitrogen (TN mg g^−1^) and phosphorous (TP mg g^−1^) within the dry-weight biomass of *Lemna minor* cultivated within an indoor, vertically stacked system.
