# Supplementary material for: Flow Rate and Water Depth Alters Biomass Production and Phytoremediation Capacity of Lemna minor
Source: Plants (Basel). 2022 Aug 21;11(16):2170. doi: 10.3390/plants11162170 (PMC9416032; doi:10.3390/plants11162170)
Supplement: Supplementary file 1 [file plants-11-02170-s001.zip › Sup Mat_Table S1_Coughlan et al..pdf]

Table S1: Water chemistry analysis for a single sample of the commercially available nutrient additives prepared using distilled water. These samples were sent to an external lab for analysis (Aquatic Services Unit, Environmental Research Institute, University College Cork, Ireland). The media were composed following the manufacturers' instructions. The Advanced Nutrients medium consisted of pH Perfect Grow (2 ml L^{-1}) and pH Perfect Micro (2 ml L^{-1}), while the General Hydroponics medium consisted of FloraGrow (0.25 ml L^{-1}) and FloraMicro (0.25 ml L^{-1}).

Water Chemistry Parameter	Advanced Nutrients	General Hydroponics
BOD (mg O_2/l)	17.30	0.23
COD (mg O_2/l)	203.00	4.56
Total Solids (g/l)	1.26	0.13
Total Nitrogen (mg N/l)	183.30	21.80
Ammonia (mg N/l)	9.13	4.32
Nitrate (mg N/l)	138.74	14.60
Nitrite (mg N/l)	<0.001	<0.001
Total Phosphorus (mg P/l)	0.87	1.89
Orthophosphate (mg P/l)	0.65	1.69
Chloride (mg Cl^-/l)	16.68	1.05
Potassium (mg K/l)	135.00	19.11
Sodium (mg Na/l)	3.20	0.39
Calcium (mg Ca/l)	112.40	7.84
Magnesium (mg Mg/l)	20.80	1.79
Iron (µg Fe/l)	1506	260
Zinc (µg Zn/l)	573	38
Copper (µg Cu/l)	67	19
Manganese (µg Mn/l)	1905	153
