# Supplementary material for: Flow Rate and Water Depth Alters Biomass Production and Phytoremediation Capacity of Lemna minor
Source: Plants (Basel). 2022 Aug 21;11(16):2170. doi: 10.3390/plants11162170 (PMC9416032; doi:10.3390/plants11162170)
Supplement: Supplementary file 1 [file plants-11-02170-s001.zip › Sup Mat_Table S2_Coughlan et al..pdf]

Table S2: Mean proportional change of nitrogen (TN mg g^{-1}) and phosphorous (TP mg g^{-1}) within the dry-weight biomass of *Lemna minor* cultivated within an indoor, vertically stacked system.

Flow rate (L min^{-1})	Depth (mm)	Nutrient content (%)		
Total nitrogen		Tray 1 (Day 7)	Tray 3 (Day 7)	Tray 5 (Day 7)
0.5	25	130.0%	131.9%	130.2%
1.5	25	115.9%	110.2%	110.0%
3.0	25	103.1%	92.9%	103.4%
0.5	50	125.0%	121.5%	130.5%
1.5	50	143.7%	137.2%	132.9%
3.0	50	116.3%	125.5%	121.6%
Total phosphorous				
0.5	25	89.2%	97.2%	94.4%
1.5	25	84.7%	82.4%	84.8%
3.0	25	84.0%	72.9%	80.1%
0.5	50	131.9%	137.4%	149.3%
1.5	50	148.9%	147.8%	140.1%
3.0	50	126.4%	141.8%	156.4%
